# Editorial: Harnessing aquatic microbial symbioses for sustainable aquaculture: unveiling biodiversity and ecosystem dynamics

**DOI:** 10.3389/fmicb.2026.1897215

**Published:** 2026-06-26

**Authors:** Marcia Saraiva, Anton Gerilovych, Hilal Ay

**Affiliations:** 1 Interdisciplinary Center for Marine and Environmental Research, University of Porto, Matosinhos, Portugal; 2 One Health Scientific and Research Institute, Kharkiv, Ukraine; 3 Institute for Problems of Cryobiology and Cryomedicine of the National Academy of Sciences of Ukraine, Kharkiv, Ukraine; 4 Department of Molecular Biology and Genetics, Faculty of Arts and Science, Yildiz Technical University, Istanbul, Türkiye

**Keywords:** aquaculture, circular economy, genomics, metagenomics, microbe-microbe and microbe-host interactions, microbial ecology, nature-based solution

The aquaculture has become one of the fastest-growing sectors of global food production and is now indispensable for ensuring food security, nutritional sustainability, and economic development. As capture fisheries face increasing pressure from overexploitation, habitat degradation, and climate change, aquaculture is expected to provide an ever-larger share of aquatic protein for a growing human population. However, the expansion of aquaculture has also been accompanied by significant challenges, including disease outbreaks, environmental deterioration, antimicrobial resistance, declining biodiversity, and increasing vulnerability to climate-related stressors. These challenges demand innovative, ecologically grounded approaches that enhance productivity while preserving ecosystem integrity.

This Research Topic brings together a diverse collection of studies that collectively demonstrate the central role of microbial communities in sustainable aquaculture. Covering finfish, crustaceans, bivalves, marine algae, and integrated production systems, these contributions highlight how microbial diversity and ecological interactions can be leveraged to improve health management, enhance environmental resilience, reduce dependence on antibiotics, and support circular bioeconomy principles.

A recurring theme throughout this Research Topic is the importance of environmental factors in shaping host–microbiome interactions. Environmental stressors, particularly those associated with climate change, are increasingly recognized as major determinants of aquaculture productivity. The study investigating *Eriocheir sinensis* larvae demonstrates how temperature profoundly influences the developmental trajectory of crustacean holobionts (Wang et al.). Reduced temperature not only impaired larval growth and metabolic performance but also reshaped microbial community composition and suppressed microbiota-associated amino acid metabolism. By integrating metabolomics and microbiome analyses, the authors reveal that environmental temperature influences host development through coordinated effects on both host physiology and microbial functionality. These findings provide valuable mechanistic insights into how climate-related fluctuations may affect larval survival and aquaculture yields.

The importance of environmental stress is further explored through investigations of microbiota responses to dissolved oxygen variability in marine invertebrates inhabiting Eastern Boundary Upwelling Systems. By comparing the microbiomes of the sessile tunicate *Pyura chilensis* and the mobile porcelain crab *Allopetrolisthes punctatus*, the study demonstrates that oxygen availability is a major driver of microbial community restructuring (Valenzuela-Muñoz et al.). The observed microbial shifts suggest potential adaptive mechanisms that may enhance host resilience under hypoxic conditions.

Environmental filtering is also highlighted in the study examining gut microbiota dynamics of *Penaeus vannamei* cultured under elevated salinity conditions. Salinity emerged as a dominant determinant of microbial community assembly, reducing diversity, altering core taxa composition, and shifting ecological processes from stochastic toward deterministic assembly mechanisms (Wang et al.). Importantly, higher salinity was associated with reduced abundance of opportunistic pathogens, suggesting that environmental parameters can profoundly influence disease susceptibility through microbiome-mediated mechanisms.

Another major theme is the growing recognition of beneficial microorganisms as active contributors to host health and disease resistance. A striking example is provided by the investigation of *Vibrio mediterranei* lineages associated with oyster larvae (Smith et al.). While members of the genus *Vibrio* are often regarded as pathogens, the authors demonstrate that specific phylogenetically distinct lineages of *V. mediterranei* confer near-complete protection against highly virulent oyster pathogens, even under elevated temperature stress. Their findings reveal that beneficial and pathogenic phenotypes are evolutionarily constrained within distinct clades that possess distinct genomic architectures.

The probiotic potential of microorganisms is also explored in shrimp aquaculture through the study of *Lactobacillus salivarius* GZPH2 (Najnine et al.). Using an impressive multi-omics approach integrating histology, microbiome profiling, mineral analyses, and proteomics, the authors demonstrate that this single-strain probiotic induces profound improvements in growth, survival, tissue regeneration, immune competence, and metabolic efficiency. Notably, the study illustrates that probiotic effects are highly strain-specific, emphasizing the need for precision probiotic selection rather than reliance on generalized microbial consortia.

In a particularly innovative contribution, researchers demonstrate that the bacteriovorous ciliate *Uronema marinum* can function as a natural biological control agent against *Vibrio* infections in bivalve hatcheries (Cés et al.). Through selective bacteriovory, the ciliate effectively reduced pathogen pressure and protected larvae at levels comparable to antibiotic treatments. This work introduces an entirely new dimension to probiotic research by highlighting the potential role of microbial predators in disease management.

The investigation of immune priming in the Manila clam (*Ruditapes philippinarum*) provides compelling evidence that enhanced disease resistance emerges not solely from host immune responses but from dynamic interactions among the host, microbiota, and pathogen (Rodino-Janeiro et al.). Primed clams exhibited dramatically improved survival following pathogen challenge while simultaneously developing distinct microbial communities characterized by beneficial bacterial taxa. Importantly, pathogens persisted at low, non-lethal levels, suggesting that protection results from ecological regulation rather than complete pathogen elimination.

Comparative genomic analysis of *Flavobacterium psychrophilum* isolated from crucian carp provides a detailed understanding of the genetic basis underlying host adaptation and pathogenicity associated with overwintering mortality syndrome (Jiang et al.). The identification of unique virulence factors, stress-response genes, and antibiotic resistance determinants underscores the importance of genomic surveillance for emerging pathogens in freshwater aquaculture systems.

Similarly, the study evaluating bacterial predation during mussel depuration highlights the complexity of manipulating the microbiome (Blaiotta et al.). Although predatory bacteria may suppress pathogenic populations, their introduction can also disrupt native microbial communities and reduce microbiome resilience. These findings serve as an important reminder that microbial interventions must be evaluated within broader ecological contexts to avoid unintended consequences.

The investigation of peanut shells as a carbon source and microbial carrier in shrimp culture systems illustrates how agricultural by-products can support environmentally sustainable aquaculture (Liu et al.). Peanut shell supplementation improved nitrogen removal while producing relatively modest perturbations to microbial community structure. Unlike rapidly degradable carbon sources, peanut shells promoted gradual enrichment of potentially beneficial bacteria without causing major ecosystem disruptions.

The study comparing conventional recirculating aquaculture systems (RAS) with integrated aquaponic systems further highlights the importance of system design in shaping microbial assembly processes (Ruiz et al.). Distinct microbial communities emerged across fish guts, biofilters, biofilms, and water compartments, reflecting habitat-specific ecological selection. Notably, aquaponic systems supported microbial taxa associated with nutrient cycling, plant growth promotion, and disease resistance, emphasizing the potential of integrated production systems to harness microbial functions for enhanced sustainability and productivity.

The growing importance of circular bioeconomy approaches is further demonstrated in the contribution exploring innovative aquafeed ingredients derived from fungi, insects, microalgae, earthworms, and aquatic plants (Girão et al.). These waste-derived biomasses exhibited diverse prebiotic, antimicrobial, antioxidant, and immunomodulatory properties. By valorizing underutilized biological resources, this research exemplifies how microbial and nutritional innovations can simultaneously improve animal health, reduce environmental impacts, and enhance resource efficiency.

The study of Atlantic Nori (*Porphyra* spp.) reveals highly structured bacterial communities associated with different developmental stages and identifies numerous bacterial taxa capable of producing auxins and other growth-promoting compounds (Cortez et al.). These findings reinforce the notion that microbial partners directly contribute to algal morphogenesis and productivity. As seaweed aquaculture continues to expand globally, microbiome-based approaches may become essential tools for optimizing cultivation success and resilience.

The comparative study of wild and captive lenok (*Brachymystax lenok*) demonstrates how environmental conditions shape intestinal morphology, immune status, antioxidant capacity, and microbiome composition (Bai et al.). Wild individuals displayed stronger immune and antioxidant responses alongside distinct microbial communities, highlighting the importance of preserving microbiome diversity in conservation and reintroduction programs. These findings suggest that microbiome-informed management strategies may improve post-release survival and ecological adaptation of endangered aquatic species.

Taken together, the studies assembled in this Research Topic reveal several overarching principles. First, microbial communities are integral components of aquatic organisms and production systems, influencing health, development, immunity, and environmental adaptation. Second, host–microbiome interactions are highly dynamic and responsive to environmental conditions, including temperature, oxygen availability, and salinity. Third, beneficial microorganisms, including bacteria, protists, and complex microbial consortia, represent powerful yet underutilized tools for disease prevention and health promotion. Fourth, sustainable aquaculture increasingly depends on ecological approaches that integrate microbial processes into management practices, production system design, and resource recycling strategies.

This Research Topic also illustrates the remarkable methodological advances transforming aquatic microbial ecology. Multi-omics technologies, network analyses, functional predictions, and high-resolution sequencing approaches now allow researchers to move beyond descriptive inventories toward mechanistic understanding.

As aquaculture enters an era characterized by climate uncertainty, intensification pressures, and growing sustainability demands, the importance of microbial symbioses will only continue to increase. Future progress will depend on translating ecological knowledge into practical applications, including precision probiotics, microbiome engineering, ecological disease management, climate-resilient production systems, and microbiome-informed conservation strategies.

We hope that this Research Topic stimulates further interdisciplinary collaboration among microbiologists, ecologists, aquaculture scientists, veterinarians, nutritionists, and systems engineers. By embracing the complexity of aquatic microbial symbioses, we can move toward a new generation of aquaculture practices that are not only productive and economically viable but also environmentally responsible and resilient.

